# Constrains for seeking perimenopausal healthcare services among women aged 40–60 years in Guangzhou, China: a cross-sectional study

**DOI:** 10.3389/fpubh.2025.1662308

**Published:** 2025-11-11

**Authors:** Yiding Wang, Yan Liu, Ribo Xiong

**Affiliations:** 1 Department of Gynecology and Obstetrics, The Third Affiliated Hospital of Southern Medical University, Guangzhou, China; 2 Department of Rehabilitation, The Seventh Affiliated Hospital, Southern Medical University, Foshan, China

**Keywords:** perimenopause, healthcare, utilization, China, menopausal symptom

## Abstract

**Introduction:**

In China, the vast majority of perimenopausal healthcare (PMH) services are performed in a diversity of outpatient clinics, such that women may encounter more obstacles toward acquiring appropriate management of perimenopause, resulting in misdiagnosis or missed diagnosis and subsequent repeated visits. Establishing a specialty clinic for perimenopausal women is a new concept. This study aimed to assess PMH services utilization among women aged 40–60 years, and identify factors influencing it's uptake in Guangzhou, China.

**Methods:**

A cross-sectional study was conducted from February to October 2024 including 1,136 women from Tianhe District, Guangzhou. Barriers and facilitators for PMH services utilization were analyzed based on Andersen's Behavioral Model of Health Services Use.

**Results:**

The magnitude of PMH services utilization was 31.0% among women aged 40–60 year in Guangzhou, China. Factors associated with services utilization included immigrant women (OR = 3.158, 95%CI:2.014–3.957), not living closer to the PMH clinic (OR = 2.841, 95%CI:1.784–3.259), women with limited decision-making autonomy regarding PMH services utilization (OR = 0.361, 95%CI:0.128–0.813), having a feeling of stigma toward perimenopausal health problems (OR = 0.284, 95%CI:0.079–0.560), poor social support (OR = 3.015, 95%CI: 1.809–3.217) and moderate/severe menopausal symptoms (OR = 2.045, 95%CI:1.610–2.967 and OR = 1.836, 95%CI:0.739–2.318, respectively).

**Discussion:**

The utilization of PMH services among women aged 40–60 years remained suboptimal. Efforts should be made to raise public concerns about perimenopause. Improving services access is critical for increasing the utilization of PMH services.

## Introduction

Perimenopause is the transitional period when a woman develops biological and clinical features around menopause up to 1 year after the last menstrual period, which mostly occurs between 40 and 60 years of age and can last for 2–10 years (1). Demographic estimates suggest that every year 25 million women experience menopause worldwide and the number of perimenopausal women is projected to reach 1.2 billion in 2030 (2). In China, there are approximately 167 million women in the perimenopausal phase according to a survey conducted by National Bureau of Statistics in 2020, accounting for 23% of the total number of perimenopausal women worldwide (3). During perimenopause, the fluctuations or decreases in sex hormones may lead to a series of ovarian hypofunction, autonomic nervous system dysfunction and neuropsychological symptoms, such as hot flashes, night sweats, fatigue, palpitation, insomnia, vaginal dryness, agitation, irritability, depression and cognitive disorders, which refers to perimenopausal syndrome (PMS) (4). These somatic and psychological symptoms can exert a negative effect on women's quality of life, and even cause family disharmony and social problems (5, 6). Given the scale of perimenopausal population, a comprehensive understanding of healthcare utilization and its determinants among this population is crucial for enhancing healthcare accessibility, promoting health development, and fostering social harmony.

In China, the majority of perimenopausal healthcare (PMH) services is performed in the outpatient clinics of public hospitals, mostly in internal medicine and gynecology clinics, the implication is that women may encounter more obstacles toward acquiring appropriate management of perimenopause, resulting in misdiagnosis or missed diagnosis and subsequent repeated visits. Establishing a specialty clinic for perimenopausal women is a new concept. In 2015, Chinese government proposed a 5-item initiative for perimenopausal women to strengthen the popularization of perimenopausal knowledge and establish PMH clinic (7). Guangzhou, where this study was conducted, is one of the pilot cities where PMH services are provided in selected public hospitals to address perimenopausal women's specific concerns and needs. Standard PMH services in China include PMS counseling; hormone therapy counseling and treatment regimen provision; psychological care; and community empowerment. Multi-disciplinary treatments and non-judgemental attitudes in service provision are developed to smooth out negative experiences and/or symptoms related to perimenopause. However, knowledge about the extent of seeking healthcare in PMH clinics is scarce. How the specific context affects the utilization of PMH services and what strategies are adopted to promote effective perimenopausal counseling continuously attract researchers' interests.

Recent studies suggest that certain socioeconomic and demographic factors may be associated with seeking healthcare among perimenopausal women (8–11). For example, employed women are less likely to go to clinics for healthcare, while women whose husbands had diseases are more likely to seek care at clinics for their menopausal symptoms (8). Other research has shown that women tend to visit hospitals when they are suffering from more than four symptoms related to perimenopause (9, 10). In addition, menstruation status, as well as severity of menopausal symptoms were also a major factor influencing the women's healthcare-seeking behavior (11). The existing literature mainly focused on determinants of seeking perimenopausal healthcare at the department of internal medicine or gynecology which has disparities in arrangements of organization, financing and delivering of care service from PMH services. Available data regarding the proportion of perimemopausal women who are willing to receive PMH services is scant and inconclusive. Moreover, factors that may be associated with PMH services utilization are not well-understood.

This study aimed to comprehensively assess PMH services utilization among women aged 40–60 years, and identify possible factors affecting its uptake based on Anderson's behavioral model in Guangzhou, China. Andersen-Nyman model is extensively referenced in health services research, particularly concerning healthcare service utilization (12). Its application in our study allows for a systematic evaluations of how individual characteristics and contextual factors influence PMH services utilization for the first time. The findings will provide evidence for improving service access and enhancing perimenopausal health.

## Materials and methods

### Study setting

This study was carried out in Tianhe District, Guangzhou, China. Guangzhou is the third largest metropolis in China with a population of more than 16 million. We chose Tianhe District because its economic and geographic development make it a good case study for China. At the end of 2023, Tianhe District had a population of 2.23 million registered residents and its GDP per capita was the highest in Guangzhou (13). In terms of healthcare reforms, the district's government always takes the lead. It was the pilot site where PMH clinics were established in three public hospitals, reflecting the cutting edge in China.

### Theoretical framework

The research is guided by Andersen-Nyman model, which provided a systematic analysis of healthcare utilization behavior by integrating social and individual factors (12). It consists of three core determinants: predisposing, enabling, and needing factors, which collectively shape the decisions to seek health services (14). Based on literature review and expert opinions, potential factors associated with the utilization of PMH services were summarized and classified into three categories. (1) Predisposing variables, such as age, parity, registered residence, education, employment, marital status and income; (2) Enabling factors, such as husband/partner's education, living arrangement, health insurance, distance to PMH clinics, family function, social support, husbands'/ partners' attitude toward receiving intervention, person who decides receiving intervention and stigma; (3) Needing factors, such as menopausal symptoms, anxiety, depression.

### Participants

A cross-sectional survey was conducted in Tianhe District of Guangzhou during the period from February to October 2024. We recruited women (1) at an age between 40 and 60 years; (2) Chinese nationality; (3) providing informed consent. We excluded those with (1) chronic diseases of the endocrine system; (2) serious psychiatric disorders or other unstable/serious comorbidity that required active treatment; (3) a history of oophorectomy or hysterectomy; (4) hormone replacement therapy within 3 months; (5) pregnant; (6) hearing impairment or cognitive dysfunction.

### Sample size

The sample size was determined assuming effect size of 0.50, α = 0.05 referring the study of Du et al. (8). To obtain reasonable estimates at 95% confidence level and 5% margin of error, considering a non-response rate of 20.0%, a total sample size of 1,050 was needed. This study included 1,143 women allowing detection of significant differences with a power of 0.88 calculated by Gpower software.

### Sampling procedure

Tianhe District has 21 sub-districts and each sub-district has several community residential committees (CRCs). Prior to the sampling procedure, we hosted a meeting with heads of CRCs, commissioners of sub-district women's federation and representatives from social work organizations on January 6th, 2024. We first introduced the aims and target population of the PMH services to the attendants. Then management of perimenopause was explained including screening for PMS, check-up, laboratory testing, treatment and follow-up. After addressing their questions, brochures detailing perimenopause and PMH services were distributed to them, and they were encouraged to spread the information.

A combination of stratified sampling and quota sampling method was adopted to select participants. First, 10 sub-districts from Tianhe District were purposefully selected in view of demographic composition and distribution, urban and suburban areas, and industry distribution. From each of 10 sub-districts, 2 CRCs were randomly selected. The eighth sub-district, located in suburban Guangzhou, is clustered with electric vehicles industry. Considering the relatively low coverage of target population in the sampling frame, 4 CRCs were randomly selected. To sum up, 22 CRCs were involved. Each CRC had a registered list of residents with their names, addresses and contacts. We first sifted out women aged 40 to 60 years, then 52 women were randomly selected from each CRC using computer-generated random numbers. The procedure of randomization was conducted by an administrative person not involved in participant recruitment. Researchers were blinded in the process of recruitment.

### Data collection

Eligible women were contacted by the investigators with the help from CRC staff. After explaining study protocol to all the women and obtaining their informal consents, participants were interviewed online, self-reported anonymous questionnaires were distributed to them through an online survey platform (‘SurveyStar', Changsha Ranxing Science and Technology, Shanghai, China.). Once the questionnaire was completed and submitted, research assistants could collect the data using the computer version of ‘SurveyStar' on the desktop computer or laptop. The data collection phase was completed with the help of 14 post-graduate students. They were trained for a day by the principal investigator covering research objectives, interview skills, quality control and research ethics. 20–30 min were needed to complete the questionnaire. Regular supervision and feedback were carried out daily by the principal researcher during the data collection period. All the completed questionnaires were checked for completeness and consistency.

### Variables and measures

#### Dependent variables

The dependent variable was utilization of PMH services. Interviewers first defined PMH as clinics in the three selected hospitals for perimenopausal women to receive comprehensive services such as screening, check-up, treatment, and counseling. The interviewer then asked: “Have you ever visited PMH clinics in the three selected hospitals?” with the option yes or no.

#### Independent variables

Demographic information was collected by a purpose-built questionnaire, including age, marital status (categorized as married, widowed or never married), employment (categorized as no /yes), education (measured in completed years of schooling), average monthly household income (categorized as 10,000–19,000, 20,000–29,000 and ≥30,000 Chinese Yuan) and health insurance (categorized as no /yes). For registered residence, we asked all participants: “Is your location of household registration in Guangzhou?” Information regarding living arrangement was collected by the question: “In your house, who are you co-residing with?” with the following optional answers: living alone, living with spouse/ children / relatives, and living with co-workers /others. Others included daughter in law, son in law, grandson/ granddaughter.

Other covariates were parity (categorized as 1 and ≥2), time spent going to the nearest perimenopausal healthcare clinic (categorized as 0–30 min and >30 min). For Husbands'/partners' attitude toward receiving PMH services, we asked participants: “How do you rate your husbands'/partners' attitude toward your visit of PMH clinic?” with the following optional answers: support, disagree, and uncertain. The decision-making authority of visiting PMH clinic was assessed by the question “In your household, who are responsible for making decisions toward your attending PMH clinic?” with the following optional answers: husband/partner, yourself, and both. We also described if women had a feeling of stigma toward perimenopausal health problems. This variable was based on responses to the question “Did you feel stigmatized toward perimenopausal health problem?” with the answers categorized as yes or no.

Family function was assessed by the Family Care Index Survey, a self-evaluation scale for the subjective and quantitative evaluation of one's family functions (15). It covers 5 domains: adaptation, partnership, growth, affection, and resolve. A total score of 7–10, 4–6 and 0–3 indicates good, moderate and severe family function, respectively (15). The Chinese version had satisfactory reliability and validity, with a Cronbach's α of 0.96 and a correlation coefficient of 0.79 (16).

Social support was measured by the Social Support Rating Scale, an instrument composed of 3 subscales (informational support, emotional support and household activity support) with a summary score which can be categorized into three levels (low, medium and high) (17). This scale showed good reliability with a Cronbach's α of 0.92 (18).

The menopausal symptoms of the participants were evaluated by the modified Kupperman menopausal index (mKMI), a questionnaire widely used internationally, and its role in clinical practice is well established (19). It includes 13 items concerning the following complaints: (1) sweating and hot flushes; (2) paresthesia; (3) insomnia; (4) nervousness; (5) melancholia; (6) vertigo; (7) fatigue; (8) arthralgia/ myalgia; (9) headache; (10) heart palpitation; (11) formication; (12) sexual complaints; (13) urinary tract infection. Each item is rated with a 4-point Likert scale: 0 (none), 1 (mild), 2 (moderate), and 3 (severe). Menopausal symptoms are considered no obvious if the total score is less than 6 points, mild if the total score is 7 to 15 points, moderate if the total score is 16 to 30 points, and severe if the total score is more than 30 points. The Chinese version of mKMI demonstrated a high level of reliability (Cronbach's alpha = 0.87) (20).

The symptoms of anxiety were gauged by the Self-Rating Anxiety Scale (SRS) (21), which had good reliability in Chinese population with a Cronbach alpha coefficient of 0.88. This scale consists of 20 items measured on a 4-point Likert scale, ranging from 1 to 4 points. The standard total SRS score is the integer part of the total SRS score of 1.25 times. If the standard total SRS score is less than 50 points, the person is considered normal with respect to anxiety. The anxious symptoms are considered mild with a standard total score of 50 to 59 points, moderate with 60 to 69 points, and severe with 70 points or more. The Self-Rating Depression Scale (SDS) was chosen to assess the severity of depression (21), which had good reliability in Chinese population with a Cronbach alpha coefficient of 0.86 (22). This scale also contains 20 items with each item assigned to 4 grades (1–4 points). The calculation method for the standard total SDS score and the evaluation criteria for the severity of depression are the same as those for SRS.

### Statistical analysis

Statistical analysis was conducted with SPSS 24.0 software. Missing outcome data were imputed by the expectation-maximization (EM) algorithm trimmed to fall between the minimum and maximum of possible values. The Chi-square test, Fisher's exact test (for categorical variables), *t*-test, and the Wilcoxon rank sum test (for continuous variables) were used to assess the differences in predisposing variables, enabling variables and needing variables between women who utilized PMH services and those who did not. Then the independent variables that were significantly associated (*P* < 0.05) with the uptake of PMH services were considered as possible contributing factors and entered into a multivariate logistic regression model. Collinearity between variables was evaluated and Collinearity between independent variables were evaluated and no significant tolerance issues were observed (Appendix 1). The Hosmer–Lemeshow goodness-of-fit test was employed to assess the predictive model which indicated a strong degree of fit (χ^2^ = 13.125*, P* = 0.107). Odds Ratios (ORs) with 95% confidence intervals (95% CIs) were calculated to measure the strength of association. Estimates with 2-sided *p*-values less than 0.05 were considered a statistically significant difference.

## Results

### Demographic characteristics of study participants

Among 1,143 women approached to participate, 1,139 (99.7%) consented to participate in the survey. Among those who consented to participate in the survey, 1,136 (99.7%) completed the interviews. Of the 1,136 women who completed the interviews, 352 (31.0%) utilized PMH services.

The average age of women was 51 years old ranging from 45 to 58 years. A majority of the participants were married and employed. 50.2% of the women reported college or higher education level. The highest proportion (39.8%) of households earned between 20,000 and 29,000 Chinese yuan per month, followed by those who earned 10,000–19,000 Chinese yuan (31.2%). Distribution by registered residence showed that more than half of the participants were internal migrants (Table 1).

**Table 1:** Demographic characteristics of study participants.

**Variables**	**Frequency**	**Percentage (%)**
Age	51.4 ± 6.7	
**Marital status**		
Married	1,062	93.5
Widowed/ Never married	74	6.5
**Employment**		
Yes	1,011	89.0
No	125	11.0
**Educational level**		
Junior high school or less	264	23.2
Senior high school	301	27.3
College or more	571	50.2
**Average monthly household income**		
10,000–19,000 CNY	354	31.2
20,000–29,000 CNY	453	39.8
≥30,000 CNY	329	29.0
**Registered residence**		
Guangzhou	512	45.1
Others	624	54.9

CNY, Chinese Yuan; 1CNY = 0.14 US dollar.

### Variations in the use of PMH services by background characteristics

The proportion of PMH services utilization was significantly lower among internal migrant women (37.2%) than among local residents (62.8%). The proportion was also significantly greater among women who lived closer to the PMH clinic (80.7%) than among those who lived far away (19.3%). In addition, the proportion was significantly greater among women who made services utilization themselves (50.9%) than among those who made the decisions jointly with their partners (35.2%) or those whose partners solely made such decisions (13.9). Similarly, the proportion that used the services was significantly higher among women who did not feel stigmatized (53.7%) than among those who had a feeling of stigma (46.3%). The proportion of services utilization was significantly higher among women with higher levels of social support (46.0%) than among women whose social support was medium (36.1%) or low (17.9%). With respect to menopausal symptoms, the severe group of mKMI (31.0%) and the moderate group of mKMI (29.0%) were significantly more likely to use the services compared with the ‘not obvious' group (13.0%). There were, however, no statistically significant variations in the proportions using the services by other factors considered (Table 2).

**Table 2:** Association of three categorical variables and utilization of perimenopausal healthcare among women aged 40–60 years in Guangzhou, China.

**Variables**	**Total [*n* (%)]**	**Perimenopausal healthcare service utilization [*****n*** **(%)]**		***x^2^*/*t***	***P***
		**No (*****n** =* **784)**	**Yes (*****n** =* **352)**		
**Predisposing factors**					
Age (year)	51.4 ± 6.7	49.6 ± 7.1	50.2 ± 9.3	12.058	0.116
Marital status				0.604	0.337
Married	1,062 (93.5)	735 (93.8)	327 (93.0)		
Widowed/Never married	74 (6.5)	49 (6.2)	25 (7.0)		
Employment				1.000	0.515
Yes	1,011 (89.0)	698 (89.0)	313 (88.9)		
No	125 (11.0)	86 (11.0)	39 (11.1)		
Educational level				1.210	0.546
Junior high school or less	264 (23.2)	175 (22.3)	89 (25.3)		
Senior high school	301 (27.3)	211 (26.9)	90 (25.6)		
College or more	571 (50.2)	398 (50.8)	173 (49.1)		
Average monthly household income				0.126	0.939
10,000–19,000 CNY	354 (31.2)	242 (30.9)	112 (31.8)		
20,000–29,000 CNY	453 (39.8)	313 (39.9)	140 (39.8)		
≥30,000 CNY	329 (29.0)	229 (29.2)	100 (28.4)		
Registered residence				0.000	0.000
Guangzhou	512 (45.1)	291 (37.1)	221 (62.8)		
Others	624 (54.9)	493 (62.9)	131 (37.2)		
Parity				0.947	0.478
1	429 (37.8)	297 (37.9)	132 (37.5)		
≥7	707 (62.2)	487 (62.1)	220 (62.5)		
**Enabling factors**					
Husbands'/Partners' education				0.047	0.977
Junior high school or less	257 (22.6)	176 (22.5)	81 (23.0)		
Senior high school	293 (25.8)	203 (25.9)	90 (25.6)		
College or more	586 (51.6)	405 (51.6)	181 (51.4)		
**Living arrangement**				0.053	0.974
Alone	296 (26.1)	203 (25.9)	93 (26.5)		
With spouse/children/relatives	525 (46.2)	364 (46.4)	161 (45.7)		
With co-workers/others	315 (27.7)	217 (27.7)	98 (27.8)		
**Health insurance**				0.792	0.413
Yes	704 (62.0)	488 (62.2)	216 (61.4)		
No	432 (38.0)	296 (37.8)	136 (38.6)		
**Time spent going to the nearest perimenopausal healthcare clinic**				0.000	0.000
0–30 min	492 (43.3)	208 (26.5)	284 (80.7)		
>30 min	644 (56.7)	576 (73.5)	68 (19.3)		
**Husbands'/partners' attitude toward receiving intervention**				0.128	0.938
Support	383 (33.7)	263 (33.5)	120 (34.2)		
Disagree	328 (28.9)	225 (28.7)	103 (29.2)		
Uncertain	425 (37.4)	296 (37.8)	129 (36.6)		
**Person responsible for making healthcare utilization decision**				61.590	0.000
Husband/partner	340 (30.0)	291 (37.1)	49 (13.9)		
Women herself	465 (40.9)	286 (36.5)	179 (50.9)		
Both	331 (29.1)	207 (26.4)	124 (35.2)		
**Self-stigma toward perimenopausal health problem**				0.003	0.002
Yes	602 (53.0)	439 (56.0)	163 (46.3)		
No	534 (47.0)	345 (44.0)	189 (53.7)		
**Family function**				0.014	0.993
Good	399 (35.1)	275 (35.1)	124 (35.2)		
Moderate	486 (42.8)	335 (42.7)	151 (42.9)		
Severe	251 (22.1)	174 (22.2)	77 (21.9)		
**Social support**					
Low	387 (34.1)	324 (41.3)	63 (17.9)	151.300	0.000
Medium	480 (42.2)	353 (45.0)	127 (36.1)		
High	269 (23.7)	107 (13.7)	162 (46.0)		
**Needing factors**					
**Menopausal symptoms**				241.900	0.000
No obvious	201	155 (19.7)	46 (13.0)		
Mild	435	340 (43.4)	95 (27.0)		
Moderate	384	282 (36.0)	102 (29.0)		
Severe	116	7 (0.90)	109 (31.0)		
**Anxiety**				0.367	0.947
No obvious	128	89 (11.4)	39 (11.1)		
Mild	406	281 (35.8)	125 (35.5)		
Moderate	504	349 (44.5)	155 (44.0)		
Severe	98	65 (8.3)	33 (9.4)		
**Depression**				0.276	0.964
No obvious	203	141 (18.0)	62 (17.6)		
Mild	539	373 (47.6)	166 (47.2)		
Moderate	329	227 (28.9)	102 (29.0)		
Severe	65	43 (5.5)	22 (6.2)		

CNY, Chinese Yuan; 1CNY, 0.14 US dollar.

### Multivariate analysis of factors associated with the use of PMH services

The results of the logistic regression model that examined the factors associated with the use of PMH services were shown in Table 3. The logistic model analysis revealed that registered residence, distance, person responsible for making services utilization decisions, stigma, social support and menopausal symptoms were significantly associated with healthcare-seeking behaviors. Women with local residence were more likely to use PMH services compared to internal migrants (OR = 3.158, 95% CI: 2.014–3.957). Similarly, women who lived closer to the PMH clinic were more likely to utilize the services compared to those who lived far away (OR = 2.841, 95% CI: 1.784–3.259). Women whose husbands were the main decision-makers regarding services use were less likely to use PMH services compared to those who made such decisions themselves (OR = 0.361, 95% CI: 0.128–0.813). Women who had a feeling of stigma were less likely to use PMH services compared to those who did not feel stigmatized (OR = 0.284, 95%CI: 0.079–0.560). In addition, women who reported high levels of social support were more likely to use PMH services (OR = 3.015, 95%CI: 1.809–3.217). The odds of PMH services utilization among women who had severe and moderate menopausal symptoms were higher than those with no obvious symptoms (OR = 2.045, 95% CI: 1.610–2.967 and OR = 1.836, 95% CI: 0.739–2.318, respectively) (Table 3).

**Table 3:** Odds ratios from multivariate logistic regression examining factors associated with perimenopausal healthcare seeking in Guangzhou, China.

**Covariates**	***OR***	***SE***	***95% CI***	***P***
**Registered residence**				
Guangzhou	3.158	0.147	2.014–3.957	0.000
Others	–	–	–	–
**Time spent going to the nearest perimenopausal healthcare clinic**				
0–30 min	2.841	0.059	1.784–3.259	0.000
>30 min	–	–	–	–
**Person responsible for making healthcare utilization decision**				
Husband/partner	0.361	0.024	0.128–0.813	0.000
Both	0.715	0.008	0.248–1.253	0.089
Women herself	–	–	–	–
**Self-stigma toward perimenopausal health problem**				
Yes	0.284	0.016	0.079–0.560	0.000
No	–	–	–	–
**Social support**				
Low	–	–	–	–
Medium	1.036	0.157	0.491–1.636	0.081
High	3.015	0.624	1.809–3.217	0.000
**Menopausal symptoms**				
No obvious	–	–	–	–
Mild	0.825	0.316	0.415–1.023	0.078
Moderate	1.836	0.504	0.739–2.318	0.016
Severe	2.045	0.327	1.610–2.967	0.000

## Discussion

This study aimed to determine the proportion of women aged 40–60 years who utilized PMH services and possible barriers to its uptake in public hospitals piloting the set-up of a specialty clinic for perimenopausal women. To our knowledge, this is one of the very few studies to examine the multifaceted factors influencing the utilization of PMH services.

The present study revealed that 31.0% of women aged 40–60 years utilized PMH services. Uptake of PMH services was comparatively higher than the 26.0% of a study conducted in Shanghai, one of the most developed cities in China (8). This disparity could be due to public finance budget to ensure its systematic implementation and multidisciplinary treatment model in the study setting. First, concerning financial resources, the present case was supported by municipal financial budget. While in the case of Shanghai, PMH clinics were fully funded by the health facilities in contrast to the government, which received limited public finance and were therefore required to generate income through services provision. Second, the present model embraced multidisciplinary care approach with specialists from different sectors. Although the importance of an integrated management approach was emphasized by hospital managers, no detailed steps were issued to ensure the systematic implementation of PMH services in the above-mentioned case. Increased workloads resulting from in-hospital referral and coordination between various sectors may hinder health professionals from providing quality services. Moreover, this kind of workload is not related to economic benefits. Because perimenopausal women often experience a diversity of physiological and psychological changes including vasomotor, somatic, cognitive, urogenital and psychological changes which vary in terms of duration, prevalence and severity (9), a multidisciplinary approach encompassing the appropriate medical specialties plays an important role in improving health outcomes and reducing healthcare costs (23). In this model, a woman was first seen by a gynecologist in a PMH clinic, where the consultation was recorded, then multidisciplinary unit involving this gynecologist and other specialists was formed through a coordinator. At each visit, this woman was seen by several specialists who made joint decisions. The well-defined structure, complete with designated roles and responsibilities for each team member, could enhance coordination and ensure every aspect of concerns is adequately addressed.

As a predisposing factor, being a local resident was significantly more likely to use PMH services compared to their internal migrant counterparts. A growing number of studies suggested immigrants usually suffer from inequalities in access to healthcare services (24, 25). Immigrants refers to the individuals who do not have permanent resident certificate and should be living over a month in the residence (26). In the past few decades, a large number of people who were originally from the poor and rural areas in western and central inland provinces migrated to southern and eastern developed regions, such as Shanghai and Guangzhou for better opportunities and income (26). Due to the fact that the utility and allocation of public resources are based on household registration policy, immigrants have much less reimbursement in medical expenses compared to the resident population (27). On the other hand, unfamiliarity with local healthcare services and health insurance policies, low health literacy and language barriers also hindered them from seeking timely medical care (24, 28). The finding suggests that local governments need to promote social integration of immigrants in order to improve access to healthcare services.

As enabling factors, distance, social support, decision-making autonomy and social support were found to be factors influencing women's healthcare-seeking behavior. Time spent going to the nearest PMH clinic in our study was associated with higher likelihood of PMH services utilization. This finding is consistent with another study that suggested distance is an important determinant of health services usage (29). This trend may stem from busy work schedules and financial pressures faced by perimenopausal women. On the other hand, women at perimenopausal age report symptoms with varied frequency. Some of these experiences last for only a short time, while vasomotor symptoms, one of the primary complaints for which women seek therapeutic management, last for average of 10 years (6). In this case, women prefer to access the services provided within a shorter walking time. The under -utilization of PMH services among those with long travel times highlights the critical impact of convenience when selecting the site of healthcare services. Another key finding of the paper was that women whose husbands were the main decision-makers regarding the utilization of PMH services were less likely to use the services compared to those who made such decisions themselves. This may be more in line with the norms of traditional Chinese family culture which favors male- domination. Opposition by the husband could be due to limited knowledge of perimenopausal conditions or financial pressure. This raised the importance of women empowerment in reproductive healthcare. We also found a significant association between stigma and the utilization of PMH services. This was consistent with findings in other qualitative studies assessing women's access to reproductive healthcare services including post-abortion care and infertility counseling (30). Women experiencing stigmatized views on perimenopausal conditions may be misled by the perception that they are neurotic and making a fuss since most symptoms are not life-threatening. Moreover, in Chinese culture, perimenopause is a derogatory word which is associated with mental disorder or neurotic, hot-tempered. This view contributed to their delays in healthcare-seeking which may worsen the situation. Additionally, middle-aged women may be hesitant to be presented at PMH clinics lest it could hurt their job promotion. Efforts should be made to address attitudes and beliefs on perimenopausal condition. Lack of social support was also associated with lower rate of healthcare-seeking behavior. This was similar to a study in Australia which concluded social support network encouraged positive help-seeking intentions and subsequent use of mental health services in older adults (31). In theoretical models, healthcare-seeking behavior mediates the relationship between social support and health (32). The hypothesis is that social support is health-promoting because it facilitates healthier behaviors in two major ways, either directly (e.g., practical support) or indirectly (e.g., social support impacts the meaning of life) (33).

As a enabling factor, the severity of menopausal symptoms was a major factor influencing women's healthcare-seeking behavior. This result was similar to a review, in which women would not seek treatment unless they experienced multiple or severe symptoms in perimenopause (8). Similar results were also found by other researchers which showed women with moderate and severe symptoms were more likely to visit hospitals compared to the women with mild or no symptoms (34). Essentially, some complaints are strongly related to poor health outcomes, leading to high demand for therapeutic management. In addition, the severity of menopausal symptoms might increase their awareness of their own health and make them see a doctor timely. In this case, a multidisciplinary treatment approach was adopted which seemed to be an appealing choice for perimenopausal women. The finding suggests the importance of individualized treatment when planning and offering care for perimenopausal women.

A few policy implications can be drawn from this study to improve PMH services. First, Low utilization rate of PMH services warrants increased attention from health authorities to improve effective use of PMH clinics. Government financing should be undertaken using a supply-side financing model, where financial support goes directly to healthcare providers. Systematic public education programs should be launched to improve women's knowledge and awareness of perimenopause. Second, PMH services should improve its coverage for immigrants. This should include enhanced education on perimenopause for immigrants, improved social integration, improved access to free guidance and counseling. Future inquiries should delve deeper into the influencers of healthcare service engagement among this demographic, aiming to foster their acceptance of essential health interventions and ensure equitable access to PMH services. Third, site selection for future PMH clinics should consider geographic locations which can maximize convenience for perimenopausal women.

There are several limitations of this study. First, the generalizability of this study would be limited as we only conducted the study in Tianhe District, where PMH services are pilotly offered. We might have missed out the proportion of women in other districts and the findings may not represent women aged 40–60 years in Guangzhou. Second, an online survey was conducted instead of face-to-face interview, which may introduce sampling bias and limit the generalizability of the findings. Third, a self-reporting questionnaire may provide response bias. Fourth, there is potential bias stemming from unmeasured confounders, such as personality and cultural background, which may impact health- seeking behaviors.

## Conclusion

This study highlighted the urgent need for comprehensive and culturally appropriate perimenopausal health services in China. To improve service utilization, targeted strategies should address the stigma on perimenopausal health and actively involve intimate partners in the promotion of PMH services utilization. Efforts should be made to raise public awareness and comprehension of perimenopausal health, cover immigrants and improve geological convenience. Further studies are needed to concentrate on how to set up a suitable long-term perimenopausal health care insurance system.

## Data Availability

The raw data supporting the conclusions of this article will be made available by the authors, without undue reservation.
