# Supplementary material for: Constrains for seeking perimenopausal healthcare services among women aged 40–60 years in Guangzhou, China: a cross-sectional study
Source: Front Public Health. 2025 Nov 11;13:1662308. doi: 10.3389/fpubh.2025.1662308 (PMC12644086; doi:10.3389/fpubh.2025.1662308)
Supplement: Supplementary file 1 [file Table_1.docx]

**Collinearity evaluation of independent variables**

|  | Unstandardized coefficient | |  | Standardized coefficient | t | P | VIF |
| --- | --- | --- | --- | --- | --- | --- | --- |
|  | β standard error | |  | β |  |  |  |
| Constant | -6.013 | 31.287 |  | - | -0.374 | 0.759 | - |
| Educational level | 1.138 | 0.130 |  | 0.625 | 13.121 | 0.057 | 1.793 |
| Marital status | 1.255 | 0.209 |  | 0.528 | 8.519 | 0.063 | 1.124 |
| Employment status | 1.752 | 1.234 |  | 0.420 | 1.847 | 0.081 | 1.706 |
| Average monthly household income | 2.136 | 0.371 |  | 1.104 | 1.559 | 0.032 | 1.185 |
| Husbands’/partners’attitude toward receiving intervention | -1.128 | 16.896 |  | -0.005 | -0.065 | 0.812 | 1.623 |

|  | Unstandardized coefficient | |  | Standardized coefficient | t | P | VIF |
| --- | --- | --- | --- | --- | --- | --- | --- |
|  | β standard error | |  | β |  |  |  |
| Constant | -3.135 | 24.187 |  | - | -0.129 | 0.841 | - |
| Educational level | 1.027 | 0.361 |  | 0.418 | 11.149 | 0.081 | 1.650 |
| Marital status | 2.321 | 0.487 |  | 0.391 | 8.359 | 0.063 | 2.135 |
| Employment status | 1.514 | 1.033 |  | 0.398 | 1.495 | 0.096 | 2.214 |
| Average monthly household income | 2.747 | 0.569 |  | 1.027 | 1.631 | 0.053 | 2.361 |
| Person responsible for making healthcare utilization decision | 0.510 | 5.563 |  | 0.010 | 0.289 | 0.007 | 1.369 |

|  | Unstandardized coefficient | |  | Standardized coefficient | t | P | VIF |
| --- | --- | --- | --- | --- | --- | --- | --- |
|  | β standard error | |  | β |  |  |  |
| Constant | -4.027 | 36.921 |  | - | -0.009 | 0.684 | - |
| Educational level | 2.136 | 0.697 |  | 0.216 | 9.614 | 0.021 | 2.158 |
| Marital status | 2.018 | 0.118 |  | 0.832 | 3.169 | 0.069 | 2.163 |
| Employment status | 1.968 | 1.153 |  | 0.169 | 1.893 | 0.037 | 3.036 |
| Average monthly household income | 3.027 | 0.692 |  | 1.053 | 1.195 | 0.099 | 1.256 |
| Self-stigma towards perimenopausal health problem | 0.759 | 9.315 |  | 0.136 | 0.646 | 0.004 | 1.418 |

|  | Unstandardized coefficient | |  | Standardized coefficient | t | P | VIF |
| --- | --- | --- | --- | --- | --- | --- | --- |
|  | β standard error | |  | β |  |  |  |
| Constant | -11.598 | 27.148 |  | - | -0.036 | 0.158 | - |
| Educational level | 3.171 | 0.036 |  | 0.147 | 9.013 | 0.022 | 1.193 |
| Marital status | 2.149 | 0.175 |  | 0.741 | 2.846 | 0.081 | 2.134 |
| Employment status | 0.960 | 1.216 |  | 0.180 | 1.423 | 0.030 | 2.569 |
| Average monthly household income | 5.127 | 0.710 |  | 1.364 | 2.056 | 0.102 | 1.262 |
| Social support | 0.863 | 12.954 |  | 0.135 | 0.501 | 0.006 | 1.317 |
